# 27-hydroxycholesterol causes cognitive deficits by disturbing Th17/Treg balance and the related immune responses in mild cognitive impairment patients and C57BL/6J mice

**DOI:** 10.1186/s12974-023-02986-5

**Published:** 2023-12-19

**Authors:** Tao Wang, Wenjing Feng, Mengwei Ju, Huiyan Yu, Zhiting Guo, Xuejing Sun, Kexin Yang, Miao Liu, Rong Xiao

**Affiliations:** School of Public Health, Beijing Key Laboratory of Environmental Toxicology, Capital Medical University, No.10 Xitoutiao, You An Men Wai, Beijing, China

**Keywords:** 27-hydroxycholesterol, Th17/Treg balance, RORγt, Amyloidosis, Cognitive decline

## Abstract

**Background:**

Cognitive impairment is associated with dysregulated immune responses. Emerging evidence indicates that Th17 cells and their characteristic cytokine-IL-17 are receiving growing interest in the pathogenesis of cognitive decline. Here, we focus on the involvement of Th17 cells in mild cognitive impairment (MCI) and the possible mechanism of cholesterol metabolite-27-hydroxycholesterol (27-OHC).

**Methods:**

100 individuals were recruited into the nested case–control study who completed cognition assessment and the detection of oxysterols and Th17-related cytokines in serum. In addition, mice were treated with 27-OHC and inhibitors of RORγt and Foxp3 (Th17 and Treg transcription factors), and the factors involved in Th17/Treg balance and amyloidosis were detected.

**Results:**

Our results showed there was enhanced 27-OHC level in serum of MCI individuals. The Th17-related cytokines homeostasis was altered, manifested as increased IL-17A, IL-12p70, IL-23, GM-CSF, MIP-3α and TNF-α but decreased IL-13, IL-28A and TGF-β1. Further, in vivo experiments showed that 27-OHC induced higher immunogenicity, which increased Th17 proportion but decreased Treg cells in peripheral blood mononuclear cells (PBMCs); Th17 proportions in hippocampus, and IL-17A level in serum and brain were also higher than control mice. The fluorescence intensity of amyloid-β (Aβ) and the precursor of amyloid A amyloidosis–serum amyloid A (SAA) was increased in the brain of 27-OHC-treated mice, and worse learning and memory performance was supported by water maze test results. While by inhibiting RORγt in 27-OHC-loaded mice, Th17 proportions in both PBMCs and hippocampus were reduced, and expressions of IL-17A and TGF-β1 were down- and up-regulated, respectively, along with a decreased amyloidosis in brain and improved learning and memory decline.

**Conclusions:**

Altogether, our results demonstrate that excessive 27-OHC aggravates the amyloidosis and leads to cognitive deficits by regulating RORγt and disturbing Th17/Treg balance.

## Introduction

Mild cognitive impairment (MCI), as the primary stage of Alzheimer’s disease (AD), is an earliest symptomatic state in which individuals suffer from greater cognitive impairment than expected for their age in a single or multiple brain domains [1]. A plethora of studies show that cholesterol homeostasis plays a significant role in cognitive decline [2]. Oxysterols are oxygenated derivatives of cholesterol. And especially, 27-hydroxycholesterol (27-OHC) is the most abundant oxysterol in the periphery, which is catalyzed by CYP27A1 and can be further metabolized to 3β-hydroxy-5-cholestenoic acid (27-CA) and 7α-hydroxy-3-oxo-4-cholestenoic acid (7-HOCA) [3, 4]. Our previous study has proved that the concentration of 27-OHC is increased in MCI patients [5]. Moreover, 27-OHC promotes amyloid-β (Aβ) accumulation by altering Aβ metabolism, resulting in the learning and memory impairment of APP/PS1 mice, while the potential mechanism deserves further exploration [6].

Evidence shows that AD individuals tend to present high levels of immunological parameters, such as various immune cells and cytokines [7, 8], which is associated with higher conversion from MCI to AD [9], and faster disease development and progression [10]. Further, it is gaining growing recognition that neuroimmune interactions have emerged as a major focus in AD [11], among which the balance of T helper 17 (Th17) and regulatory T (Treg) cells in central nervous system and peripheral circulation has been considered to be the etiological link in this disease [12]. Remarkably increased Th17-related cytokines, such as IL-17 and IL-21, were observed in the hippocampus, cerebrospinal fluid and serum of AD patients, while decreased IL-10 and IL-35, the immunosuppressive cytokines produced by Treg cells were detected [13], suggesting that the balance between the two subpopulations exerts functions to prevent excessive immune activation and maintain immune homeostasis. 27-OHC is a potent agonist of retinoic acid-related orphan receptor γt (RORγt), which is specifically required for the differentiation and functionality of Th17 cells [14, 15], while it is still unclear whether 27-OHC participating in cognitive decline by regulating RORγt and Th17-related immune responses.

Besides, Th17 signaling could be promoted by serum amyloid A proteins (SAA), the precursor of reactive amyloid A (AA) amyloidosis, the possible mechanism of which might be due to SAA acting on poised RORγt and inducing the differentiation and migration of Th17 cells [16, 17]. Increased SAA caused by abnormal pathology induces the formation of AA amyloid, which is similar to the way Aβ involved in AD onset [18]. Considering that SAA is lipophilic and related to cholesterol metabolism, there is a possibility for SAA to be regulated by 27-OHC [19]. Therefore, it seems to be a kind of association among 27-OHC, Th17 signaling and amyloidosis, while the underlying mechanisms remain largely unsettled.

As mentioned above, we hypothesized that the alteration of 27-OHC and Th17-related cytokines is associated with cognitive decline in older adults. The underlying mechanism might be that 27-OHC interferes with Th17/Treg balance by regulating RORγt, aggravates the amyloidosis and induces learning and memory impairment. To validate these assumptions, we carried out a nested case–control study. Furtherly, in vivo experiments on C57BL/6J mice were conducted to explore the molecular mechanisms.

## Materials and methods

### Human subjects

Human participants were recruited from a multicenter study of community-dwelling subjects aged 50–70 years in China. According to inclusion and exclusion criteria described elsewhere [20], 48 MCI individuals and 52 matched (age ± 5 years, sex and education) controls were selected. Individuals with diseases known to impair cognition (e.g. brain trauma, depression) or other severe diseases (e.g. malignant tumors, cardiac failure) and those taking certain drugs (e.g. statins, immunotherapy drugs) were excluded. Montreal Cognitive Assessment (MoCA) and Mini-Mental State Examination (MMSE) [21] were used by neurologists to diagnose MCI. Face-to-face interviews were performed to collect demographic characteristics, lifestyle and clinical history of chronic diseases. Fast blood was collected and immediately centrifuged to collect serum, and then stored in − 80 ℃ until detected. This study was conducted with approvement of the Ethics Committee of Capital Medical University (2013SY35) and all participants have signed the informed consent before inclusion.

Detection of biological indicators: The concentration of oxysterols in serum was detected by HPLC–MS as described in our previous study [22]. The sandwich-based antibody microarray was used to detect Th17-related cytokines in serum. Specifically, the antibody microarray underwent a series of processing including antibody blocking, biotinylated antibody incubation and Cy3 equivalent dye–streptavidin incubation, followed by fluorescence detection (InnoScan 300 Microarray Scanner, wavelength = 532 nm, resolution = 10 μm).

### Animals and experimental design

A total of sixty 9-month-old male C57BL/6J mice were purchased from Beijing SPF Biotechnology Co., LTD. The mice were raised in the animal center of Capital Medical University under the control of light (12 h light/dark cycle), humidity (50–55%), temperature (20–23 ℃) with standard diet and water ad libitum. These mice were randomly divided into six groups (n = 10 mice/group) after a 2-week adaptation, named Control (saline 0.2 mL/day, s.c., qd for 21 days), 27-OHC (5.5 mg/kg, s.c., qd for 21 days), SR1001 (RORγt inhibitor, 25 mg/kg, i.p., bid for 18 days), P60 (forkhead box protein P3 (Foxp3) inhibitor, 50 nmol per mouse, i.p., qod for 10 times), 27-OHC + SR1001 and 27-OHC + P60 group. The neurobehavioral tests were conducted after the above treatments. Then, the mice were anesthetized and euthanized to collect blood samples and fresh tissues. The samples were weighted and frozen at − 80 ℃ until detected. All animal experiments were approved by the Ethics Committee of Capital Medical University (Ethics: AEEI-2014-047).

### Neurobehavioral tests

#### Novel object recognition test

Novel object recognition test was used to assess the short-term/working memory as described previously [23]. In brief, mice were brought to an experimental box with two identical objects placed as the familiar objects. The mice were put into the box from a certain position to explore the two objects for 10 min. One hour later, replacing one of the familiar objects with a different one, mice were released into the box from the same position to explore for another 10 min. 75% ethanol solution was used to mask the odor cues after each operation. The frequency and time of exploring novel/familiar objects were recorded and the indexes (novel object recognition index (NORI), frequency identification index (FDI), time discrimination index (TDI)) were calculated.

#### Morris water maze (MWM) test

The MWM test was performed to assess the spatial learning and memory capability. In brief, an escape platform was submerged in a pool filled with white water (dyeing by titanium dioxide powder, 21 ± 1 ℃) and located in the center of southeast quadrant. Five-day orientation navigation tests were conducted four times per day. The mice were allowed to swim for 90 s to search for the escape platform and would be guided to and stay for 15 s if they did not find it. On the sixth day, each mouse was released from the northwest quadrant to swim for 90 s with the platform removed. Finally, all results for each mouse were recorded and calculated individually.

### Histopathological analysis

HE staining: The whole brains were fixed with 4% paraformaldehyde for 24 h and embedded in paraffin. Tissues were sectioned to about 5 μm thickness, dewaxed, and then stained with Hematoxylin solution for 3–5 min, Eosin dye for 5 min, dehydrated, and ultimately sealed with neutral gum. Prepared sections were scanned and analyzed with an automatic section scanner.

Immunohistochemical staining: After dewaxed, the prepared paraffin sections were preprocessed in citrate buffer (pH 6.0) to restore antigen and 3% hydrogen peroxide to inhibit the endogenous peroxidase activity, and then bound with 3% BSA. The sections were incubated with primary antibody overnight at 4 ℃, followed by horseradish peroxidase-labeled secondary antibody for 50 min at room temperature. Afterward, the sections were developed by DAB, counterstained by hematoxylin, dehydrated, mounted and scanned for analysis.

Immunofluorescence staining: After dewaxed, the prepared paraffin sections were preprocessed in EDTA Antigen Retrieval Buffer (pH 8.0) and PBS (pH 7.4), then bound with 3% BSA. The sections were subsequently incubated with primary antibody overnight at 4 ℃, followed by secondary antibody for 50 min at room temperature in the dark. After which, DAPI and autofluorescence quencher were added successively, finally the anti-fluorescence quencher was used to mount the sections for analysis.

### Flow cytometry

Flow cytometry was performed to assess the proportions of Th17 and Treg cells in hippocampus and peripheral blood mononuclear cells (PBMCs). In brief, separated hippocampus tissue was cut and digested, then blown away into single cells. An isolation kit of mononuclear cells in mice (Tianjin Haoyang, Tianjin, China) was used to prepare suspensions of PBMCs. Then single cell suspensions were incubated with the antibodies of CD4-FITC (Proteintech, Chicago, USA) and CD25-APC (Proteintech) in the dark at 4 ℃ for 40 min according to the manufacturer's protocol. Subsequently, intracellular staining of IL-17A-PE (Invitrogen, California, USA) and Foxp3-PE-Cyanine7 (Invitrogen) was conducted in the dark at 4 ℃ for 8 h after fixation and permeabilization. At last, a Novocyte flow cytometer (Agilent, California, USA) was used to acquire the stained cell images. Analysis was performed with the NIS-Elements Viewer v5.21software (Nikon, Tokyo, Japan).

### High-performance liquid chromatography–mass spectrometry (HPLC–MS)

The concentration of 27-OHC and 24S-hydroxycholesterol (24S-OHC) in the brain and serum was measured by HPLC–MS. In brief, 50 μl brain homogenate or serum were prepared along with 50 μl d5-27-OHC and d7-24-OHC as an internal standard in it. Then the samples were subjected to acidic hydrolysis and derivatization, followed by evaporated in a vacuum dryer. The contents were redissolved in 1 mL methanol and further centrifugated at 13,000 rpm for 10 min. Finally, the supernatants were collected and analyzed by HPLC–MS.

### Quantitative real-time PCR (RT-qPCR)

Total mRNA was isolated from the brain and liver by SV Total RNA Isolation system (Promega Corporation, Madison, Wisconsin, USA). The concentration of mRNA was detected by RT-qPCR including RORγt, Foxp3, IL-17A, granulocyte–macrophage colony-stimulating factor (GM-CSF), macrophage inflammatory protein 3α (MIP-3α), IL-10, transforming growth factor β1 (TGF-β1), interferon-λ2 (IFN-λ2), CYP27A1, CYP7B1, amyloid precursor protein (APP) and SAA. In brief, 1 µg of total RNA of each sample was added into the First Strand cDNA Synthesis Kit (ThermoFisher Scientific, Waltham, MA, USA). The primers were designed specifically by retrieving from the NCBI (Table 1). RT-qPCRs were conducted using KAPA SYBR® PCR Kit (Kapa Biosystems, Woburn, MA, USA) with GAPDH as an internal reference for normalization. Each sample was performed at least in triplicate from three biological replicas.

**Table 1:** Primers used in this study

Primer	Forward sequence (5'-3')	Reverse sequence (5'-3')
RORγt	ACAAATTGAAGTGATCCCTTGC	GGAGTAGGCCACATTACACTG
Foxp3	TTTCACCTATGCCACCCTTATC	CATGCGAGTAAACCAATGGTAG
IL-17A	GAGCTTCATCTGTGTCTCTGAT	GCCAAGGGAGTTAAAGACTTTG
GM-CSF	TTCAAGAAGCTAACATGTGTGC	GGTAACTTGTGTTTCACAGTCC
MIP-3α	TCTTCCTTCCAGAGCTATTGTG	GACTGCTTGTCCTTCAATGATC
IL-10	TTCTTTCAAACAAAGGACCAGC	GCAACCCAAGTAACCCTTAAAG
TGF-β1	CCAGATCCTGTCCAAACTAAGG	CTCTTTAGCATAGTAGTCCGCT
IFN-λ2	GGATTGCCACATTGCTCAGTTCAAG	GTCCTTCTCAAGCAGCCTCTTCTC
CYP27A1	ATCGCACAAGGAGAGCAATGGTAC	GGCAAGGTGGTAGAGAAGATGAGC
CYP7B1	AACCCTTTCCAGTACCAGTATG	GTGAACGTCTTCATTAAGGTCG
APP	TGAATGTGCAGAATGGAAAGTG	AACTAGGCAACGGTAAGGAATC
SAA	ACACTGACATGAAGGAAGCTAA	CCTTTGAGCAGCATCATAGTTC
GAPDH	GGTTGTCTCCTGCGACTTCA	TGGTCCAGGGTTTCTTACTCC

RORγt: retinoic acid-related orphan receptor γt; Foxp3: forkhead box protein p3; IL: Interleukin; GM-CSF: granulocyte–macrophage colony-stimulating factor; MIP-3α: macrophage inflammatory protein 3α; TGF-β1: transforming growth factor β1; IFN-λ2: Interferon-λ2; CYP27A1: sterol 27-hydroxylase; CYP7B1: oxysterol 7alpha-hydroxylase; APP: amyloid precursor protein; SAA: serum amyloid A

### Western blot

Approximately 40 mg brain or liver tissue were lysed in RIPA buffer including protease inhibitors. Then the tissues were homogenized and centrifuged (12,000 rpm, 5 min, 4 ℃) to collect the supernatant. The concentration of protein was measured by bicinchoninic acid (BCA) method. 40 µg protein samples were separated by 12.5% SDS-PAGE and transferred to PVDF membranes. The antibodies used were as below: RORγt 1:2000 (Abcam, ab207082), Foxp3 1:1000 (Abcam, ab215206), IL-17A 1:3000 (Abcam, ab189377), GM-CSF 1:1000 (Proteintech, 17762-1-AP), MIP-3α 1:1000 (Abcam, ab106151), IL-10 1:1000 (Abcam, ab189392), TGF-β1 1:1000 (Abcam, ab179695), IFN-λ2 0.1 µg/mL (R&D, AF4635), CYP27A1 1:1000 (Abcam, ab126785), CYP7B1 1:1000 (ABclonal, A17872), APP 1:1000 (Abcam, ab126732), and SAA 1:1000 (Abcam, ab199030). Image System Fusion FX (Vilber Lourmat, Paris, France) was used to detect the protein density.

### Statistical analysis

Data analysis was processed using SPSS 23.0 and GraphPad Prism 8.0.1 software. Demographic characteristics were displayed as mean ± standard deviation (SD) or median (interquartile range) in accordance with the sample distribution. Parametric or nonparametric data were analyzed by Student’s t test or the Mann–Whitney test, respectively. Qualitative data were shown as a percentage and compared with the Chi-square test. The Spearman correlation was used to assess the correlations between data. In animal studies, differences among groups were evaluated with one-way analysis of variance (ANOVA) and post-hoc comparisons were conducted with LSD-t OR Dunnett T3 test. Repeated measurement analysis of MWM tests was assessed by Two-way ANOVA. All statistical analyses were 2-sided and statistical differences were considered when *p* < 0.05.

## Results

### Nested case–control study

#### Demographic and clinical characteristics

Demographic characteristics are tabulated in Table 2. It showed that the differences of MMSE and MoCA scores between MCI patients and controls were significant. MCI patients presented worse MMSE and MoCA scores (*p* < 0.001) than the controls. No significant difference was observed in other characteristics such as body mass index (BMI), prevalence rate of chronic diseases, education years, drinkers and exercise.

**Table 2:** Demographic characteristics of MCI and control participants

	MCI (*n* = 48)	Control (n = 52)	*t*/Z/χ2	*p* value
Demographic characteristics				
Age	62 (58, 66.75)	63.5 (61, 65)	− 0.956	0.339
Male, *n* (%)	22 (45.8%)	24 (46.2%)	0.001	0.974
Education years	9 (9, 12)	9 (9, 12)	− 0.099	0.921
Current smoker, *n* (%)	16 (33.3%)	8 (15.4%)	4.408	0.036*
Current drinker, *n* (%)	17 (35.4%)	15 (28.8%)	0.495	0.482
Exercise, *n* (%)	35 (72.9%)	42 (80.0%)	0.869	0.351
BMI, kg/m^2^	24.65 ± 3.15	24.30 ± 3.08	0.559	0.578
Hypertension, *n* (%)	17 (35.4%)	19 (36.5%)	0.014	0.907
Diabetes, *n* (%)	7 (14.6%)	8 (15.4%)	0.013	0.911
Hyperlipidemia, n (%)	19 (39.6%)	19 (36.5%)	0.098	0.754
Atherosclerosis, n (%)	5 (10.4%)	4 (7.7%)	0.016	0.900
CHD, *n* (%)	5 (10.4%)	3 (5.8%)	0.237	0.626
MCI screening				
MMSE scores	27 (26, 28)	29 (28, 30)	− 4.868	< 0.001
MoCA scores	22 (19.25, 23.75)	27 (25.25, 28)	− 7.629	< 0.001

*p < 0.05. BMI: body mass index; CHD: coronary heart disease; MMSE: Mini-Mental State Examination; MoCA: Montreal Cognitive Assessment

### Level of oxysterols and Th17-related cytokines in the serum

The changes of four oxysterols in serum of MCI and control subjects were detected. As shown in Table 3, higher levels of 27-OHC (*p* = 0.030) and 27-OHC/24S-OHC (*p* = 0.015) were found in MCI patients. However, there were no significant difference in 24S-OHC, 27-CA, 7-HOCA and 27-OHC/7-HOCA (*p* > 0.05) between the two groups. There were significant differences in the levels of nine Th17-related cytokines between the two groups, in which the concentrations of IL-17A, IL-12p70, IL-23, GM-CSF, MIP-3α and TNF-α were increased, while IL-13, IL-28A and TGF-β1 were decreased in MCI patients compared with control subjects.

**Table 3:** Comparison of serum oxysterols and Th17-related cytokines between MCI and control participants

	MCI (*n* = 48)	Control (*n *= 52)	t/Z	*p* value
Oxysterols				
27-OHC, μmol/L	1.343 (0.650, 2.238)	0.961 (0.275, 1.485)	− 2.164	0.030*
24S-OHC, μmol/L	4.007 ± 1.103	4.102 ± 0.881	− 0.469	0.641
27-CA, μmol/L	0.147 (0.105, 0.192)	0.146 (0.086, 0.193)	− 0.267	0.790
7-HOCA, μmol/L	0.034 (0.025, 0.046)	0.036 (0.025, 0.047)	− 0.441	0.659
27-OHC/24S-OHC	0.374 (0.165, 0.645)	0.249 (0.070, 0.382)	− 2.421	0.015*
27-OHC/7-HOCA	40.018 (16.364, 64.046)	28.821 (9.203, 42.739)	− 1.508	0.132
Th17-related cytokines				
IL-17A	3.662 (2.575,5.359)	3.191 (2.154,3.992)	− 2.360	0.018*
IL-12p70	0.530 (0.277,0.742)	0.365 (0.249,0.522)	− 2.791	0.005*
IL-23	79.220 (46.325,109.336)	57.645 (29.665,82.986)	− 2.632	0.008*
GM-CSF	5.626 (4.360, 7.702)	5.596 (3.396,6.703)	− 1.979	0.048*
MIP-3α	10.607 (3.590,30.401)	6.840 (2.515,16.891)	− 2.212	0.027*
TNF-α	13.931 (8.822,18.382)	11.019 (7.840,15.733)	− 2.308	0.021*
IL-13	1.699 ± 0.973	2.369 ± 1.622	− 2.484	0.017*
IL-28A	0.000 (0.000,0.175)	0.000 (0.000,11.357)	− 2.004	0.045*
TGF-β1	193.192 (79.751,301.129)	245.234 (141.176,401.015)	− 2.201	0.028*
IFN-γ	8.525 ± 3.047	8.659 ± 5.537	− 0.140	0.889
IL-2	10.676 ± 5.527	11.212 ± 8.925	− 0.346	0.731
IL-5	5.492 ± 2.378	6.252 ± 3.477	− 1.225	0.227
TNF-β	9.430 ± 10.378	6.639 ± 2.468	1.958	0.056
IL-6	9.302 (6.199, 14.979)	8.357 (5.483, 13.092)	− 0.967	0.334
IL-10	0.821 (0.598,1.034)	0.720 (0.594, 0.931)	− 0.921	0.357
IL-17F	0.231 (0.065,0.786)	0.117 (0.008,0.569)	− 1.398	0.162
IL-1β	1.039 (0.376,2.286)	1.414 (0.758,2.342)	− 0.603	0.546
IL-4	10.122 (6.701,16.334)	11.388 (9.142,12.783)	− 0.525	0.600
IL-21	316.160 (257.939,425.695)	320.318 (254.223,434.132)	− 0.582	0.561
IL-22	0.000(0.000,83.291)	0.000(0.000,90.544)	− 0.299	0.765

*p < 0.05. 27-OHC: 27-hydroxycholesterol; 24S-OHC: 24S-hydroxycholesterol; 27-CA: 3β-hydroxy-5-cholestenoic acid; 7-HOCA: 7α-hydroxy-3-oxo-4-cholestenoic acid; IL: Interleukin; GM-CSF: granulocyte–macrophage colony-stimulating factor; MIP-3α: macrophage inflammatory protein 3α; TNF-α: tumor necrosis factor-α; TGF-β1: transforming growth factor β1; IFN-γ: Interferon-γ; TNF-β: tumor necrosis factor-β

### Correlation of Th17-related cytokines and cognition assessments

Spearman correlation was conducted to evaluate the correlation between Th17-related cytokines and cognition, the results are shown in Table 4. In detail, there were significant negative correlations between serum IL-17A with MMSE scores (*r* = − 0.202, *p* = 0.045) and MoCA scores (*r* = − 0.227, *p* = 0.024). The same results were found in MIP-3α (MMSE: *r* = − 0.231, *p* = 0.022; MoCA: *r* = − 0.241, *p* = 0.016). On the contrary, the IL-13 level was positively correlated with MoCA scores (*r* = 0.207, *p* = 0.040).

**Table 4:** Correlations between serum Th17-related cytokines and cognitive assessments

Th17-related cytokines	MMSE		MoCA	
	*r*	*p* value	*r*	*p* value
IL-17A	− 0.202	0.045*	− 0.227	0.024*
IL-12p70	− 0.098	0.338	− 0.137	0.182
IL-23	− 0.036	0.730	− 0.018	0.861
GM-CSF	0.046	0.650	− 0.104	0.306
MIP-3α	− 0.231	0.022*	− 0.241	0.016*
TNF-α	− 0.144	0.154	− 0.151	0.133
IL-13	0.153	0.130	0.207	0.040*
IL-28A	0.023	0.823	0.053	0.598
TGF-β1	0.065	0.524	0.118	0.243
IL-2	− 0.013	0.899	− 0.040	0.697
IL-5	0.039	0.700	0.037	0.714
TNF-β	− 0.136	0.177	− 0.132	0.190
IL-6	0.002	0.984	− 0.010	0.922
IL-10	− 0.121	0.235	− 0.119	0.239
IFN-γ	− 0.084	0.406	− 0.085	0.402
IL-17F	− 0.069	0.511	− 0.113	0.284
IL-1β	0.023	0.823	0.044	0.668
IL-4	− 0.121	0.241	− 0.002	0.984
IL-21	− 0.001	0.995	0.051	0.618
IL-22	0.060	0.555	0.067	0.511

*p < 0.05. IL: Interleukin; GM-CSF: granulocyte–macrophage colony-stimulating factor; MIP-3α: macrophage inflammatory protein 3α; TNF-α: tumor necrosis factor-α; TGF-β1: transforming growth factor β1; IFN-γ: Interferon-γ; TNF-β: tumor necrosis factor-β

### Logistic regression of Th17-related cytokines and the risk of MCI

**Fig. 1:**
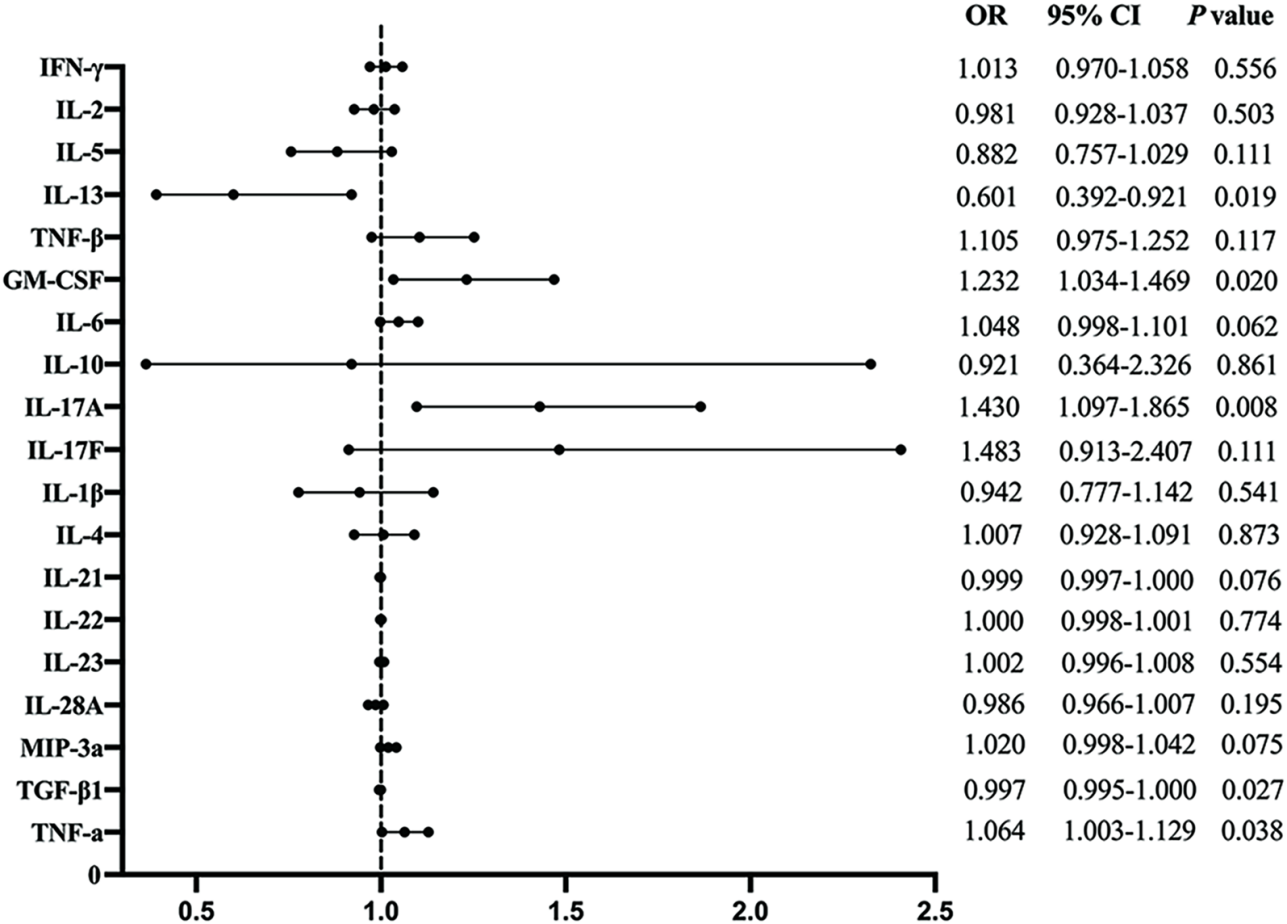
Logistic regression of Th17-related cytokines in serum and the risk of MCI. Correlation analyses were conducted by Spearman correlation coefficient. ∗*p* < 0.05

As shown in Table 5 and Fig. 1, serum IL-17A (OR = 1.430, 95% CI 1.097–1.865, *p* = 0.008), IL-12p70 (OR = 9.644, 95% CI 1.954–47.604, *p* = 0.005), GM-CSF (OR = 1.232, 95% CI 1.034–1.469, *p* = 0.020), and TNF-α (OR = 1.064, 95% CI 1.003–1.129, *p* = 0.038) could significantly increase the risk of MCI, while IL-13 (OR = 0.601, 95% CI 0.392–0.921, *p* = 0.019) and TGF-β1 (OR = 0.997, 95% CI 0.995–1.000, *p* = 0.027) decreased the risk of MCI.

**Table 5:** Logistic regression analysis between Th17-related cytokines and MCI risk

Th17-related cytokines	OR	95% CI	*p* value
IL-17A	1.430	1.097–1.865	0.008*
IL-12p70	9.644	1.954–47.604	0.005*
IL-23	1.002	0.996–1.008	0.554
GM-CSF	1.232	1.034–1.469	0.020*
MIP-3α	1.020	0.998–1.042	0.075
TNF-α	1.064	1.003–1.129	0.038*
IL-13	0.601	0.392–0.921	0.019*
IL-28A	0.986	0.966–1.007	0.195
TGF-β1	0.997	0.995–1.000	0.027*
IFN-γ	1.013	0.970–1.058	0.556
IL-2	0.981	0.928–1.037	0.503
IL-5	0.882	0.757–1.029	0.111
TNF-β	1.105	0.975–1.252	0.117
IL-6	1.048	0.998–1.101	0.062
IL-10	0.921	0.364–2.326	0.861
IL-17F	1.483	0.913–2.407	0.111
IL-1β	0.942	0.777–1.142	0.541
IL-4	1.007	0.928–1.091	0.873
IL-21	0.999	0.997–1.000	0.076
IL-22	1.000	0.998–1.001	0.774

*p < 0.05. IL: Interleukin; GM-CSF: granulocyte–macrophage colony-stimulating factor; MIP-3α: macrophage inflammatory protein 3α; TNF-α: tumor necrosis factor-α; TGF-β1: transforming growth factor β1; IFN-γ: Interferon-γ; TNF-β: tumor necrosis factor-β

Overall, there was significant correlation between higher 27-OHC and cognitive decline in MCI patients. Besides, the Th17-related cytokines spectrum of MCI patients altered significantly, including increased immuno-promoting cytokines and decreased immuno-suppressive cytokines, which showed significant correlation with cognitive decline. However, the mechanism is not yet clear. On this basis, animal study was carried out to explore the possible mechanism of cognitive impairment caused by 27-OHC through inappropriate immune responses.

### Animal study

#### Effects of experimental intervention on learning and memory ability

**Fig. 2:**
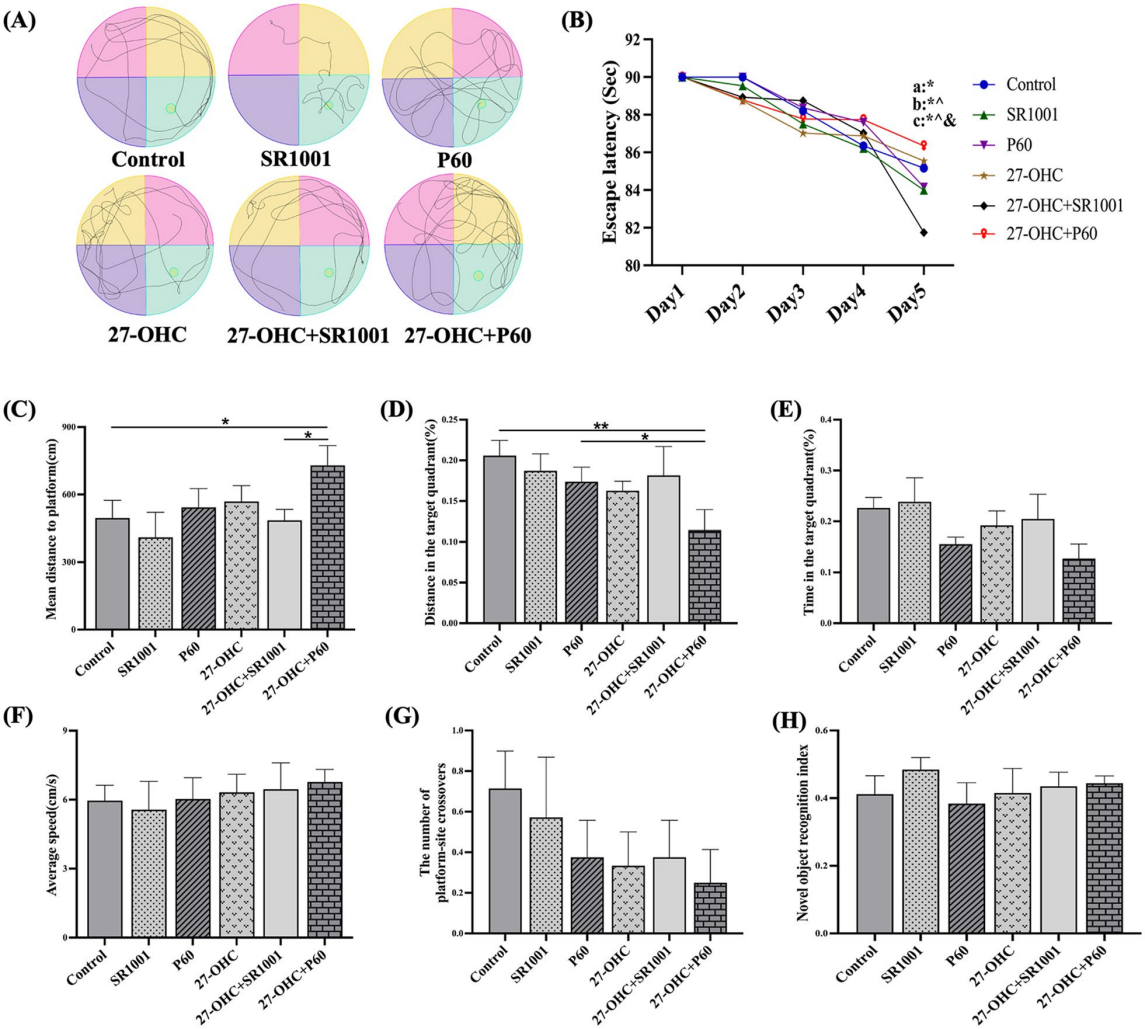
Effects of experimental intervention on novel object recognition and MWM test (*n* = 10 mice/group). **A** Swimming path; **B** escape latency, a*: SR1001 group, Day 1 vs Day 5, *p* = 0.038; b*: P60 group, Day 1 vs Day 5, *p* = 0.019, b^: P60 group, Day 2 vs Day 5, *p* = 0.019; c*: 27-OHC + SR1001 group, Day 1 vs Day 5, *p* = 0.003, c^: 27-OHC + SR1001 group, Day 2 vs Day 5, *p* = 0.003, c&: 27-OHC + SR1001 group, Day 3 vs Day 5, *p* = 0.010; **C** mean distance to platform; **D** distance in the target quadrant (%); **E** time in the target quadrant (%); **F** average speed; **G** the number of platform-site crossovers; **H** novel object recognition index (NORI) = Fn/Tn × 100% (Fn: exploring frequency of novel object; Tn: exploring time of novel object). All data are presented as mean ± SD. *p < 0.05. **p < 0.01

The MWM and novel object recognition test were carried out to evaluate the learning and memory ability of mice. In the MWM test (Fig. 2A–G), with the 5-day training, the escape latency of SR1001 and 27-OHC + SR1001 mice in Day 5 were significant decreased than that in Day 1 (*p* = 0.038, *p* = 0.003), and the results of 27-OHC + SR1001 group in Day 5 were also decreased compared with Day 2 (*p* = 0.008) and Day 3 (*p* = 0.010). The mean distance to platform in 27-OHC + P60 group was significantly longer than that in the control (*p* = 0.047) and 27-OHC + SR1001 (*p* = 0.047) group. The distance in target quadrant was evidently shortened in all the three 27-OHC-treated groups, although significant difference was only observed in 27-OHC + P60 group (control: *p* = 0.009, P60: *p* = 0.043). In addition, the number of platform-site crossovers and time in target quadrant in 27-OHC group showed a decreasing trend, which were slightly higher in the 27-OHC + SR1001 group. The NORI showed the same trend as the results of MWM test, although there was no significant difference among the groups (Fig. 2H and Table 6).

**Table 6:** FDI and TDI values of mice after intervention (*n* = 10 mice/group)

Groups	FDI	TDI
Control	− 0.048 ± 0.121	− 0.102 ± 0.500
SR1001	− 0.031 ± 0.203	− 0.096 ± 0.201
P60	− 0.108 ± 0.167	− 0.063 ± 0.245
27-OHC	− 0.027 ± 0.121	− 0.022 ± 0.367
27-OHC + SR1001	− 0.101 ± 0.226	0.022 ± 0.282
27-OHC + P60	0.028 ± 0.194	0.080 ± 0.151
F value	0.638	0.407
*p* value	0.672	0.841

Frequency identification index (FDI) = (Fn − Ff)/(Fn + Ff), time discrimination index (TDI) = (Tn − Tf)/(Tn + Tf) (Fn: exploring frequency of novel object; Tn: exploring time of novel object; Ff: exploring frequency of familiar object; Tf: exploring time of familiar object); SR1001: inhibitor of RORγt; P60: inhibitor of Foxp3; 27-OHC: 27-hydroxycholesterol

### Effects of experimental intervention on Th17/Treg balance and the immunomodulatory factors

**Fig. 3:**
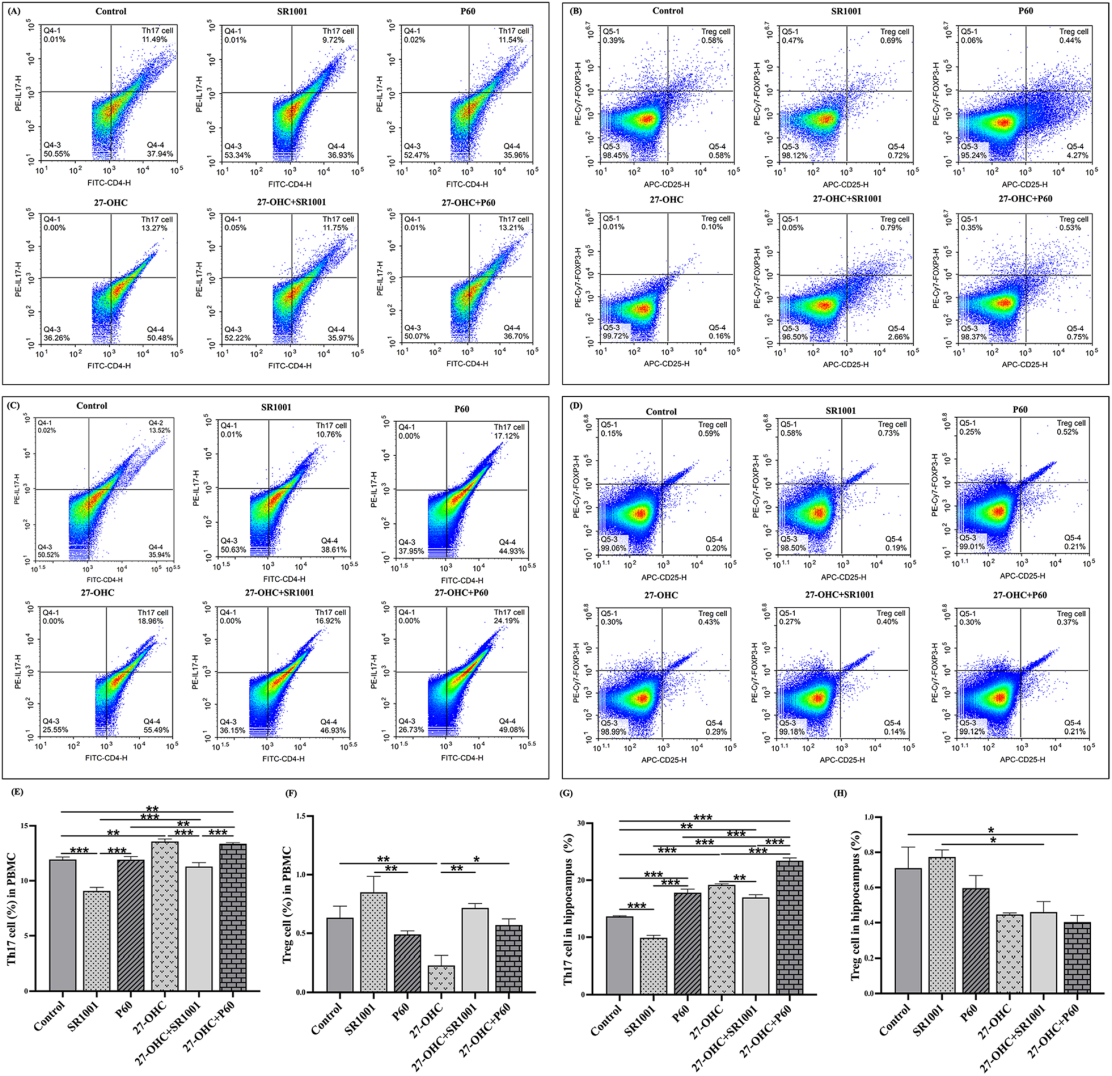
Proportions of Th17/Treg cells in PBMCs and hippocampus (%). **A**–**D** Flow cytometry results of Th17 cells in PBMCs, Treg cells in PBMCs, Th17 cells in hippocampus and Treg cells in hippocampus. **E**–**H** Statistical results of Th17 cells in PBMCs, Treg cells in PBMCs, Th17 cells in hippocampus and Treg cells in hippocampus. All data are presented as mean ± SD. *p < 0.05. **p < 0.01. ***p < 0.001

First, flow cytometry was used to directly evaluate the proportions of Th17 and Treg cells after the mixed treatment (Fig. 3). Compared with the control group, the ratio of Th17 cells in 27-OHC group was significantly increased in both PBMCs (*p* = 0.001) and hippocampus (*p* < 0.001), but the Treg cells ratio in PBMCs was decreased (*p* = 0.004). By inhibiting RORγt, mice in SR1001 group showed lower proportion of Th17 cells compared with the control group (PBMCs: *p* < 0.001, hippocampus: *p* < 0.001), while mice in P60 group showed increased Th17 cells (PBMCs: *p* < 0.001, hippocampus: *p* < 0.001) but decreased Treg cells (PBMCs: *p* = 0.009) proportions compared to SR1001 group. In addition, proportion of Th17 cells in 27-OHC + SR1001 group was higher than that of SR1001 group (PBMCs: *p* < 0.001, hippocampus: *p* < 0.001), but lower than the 27-OHC group (PBMCs: *p* < 0.001, hippocampus: *p* = 0.005).

**Fig. 4:**
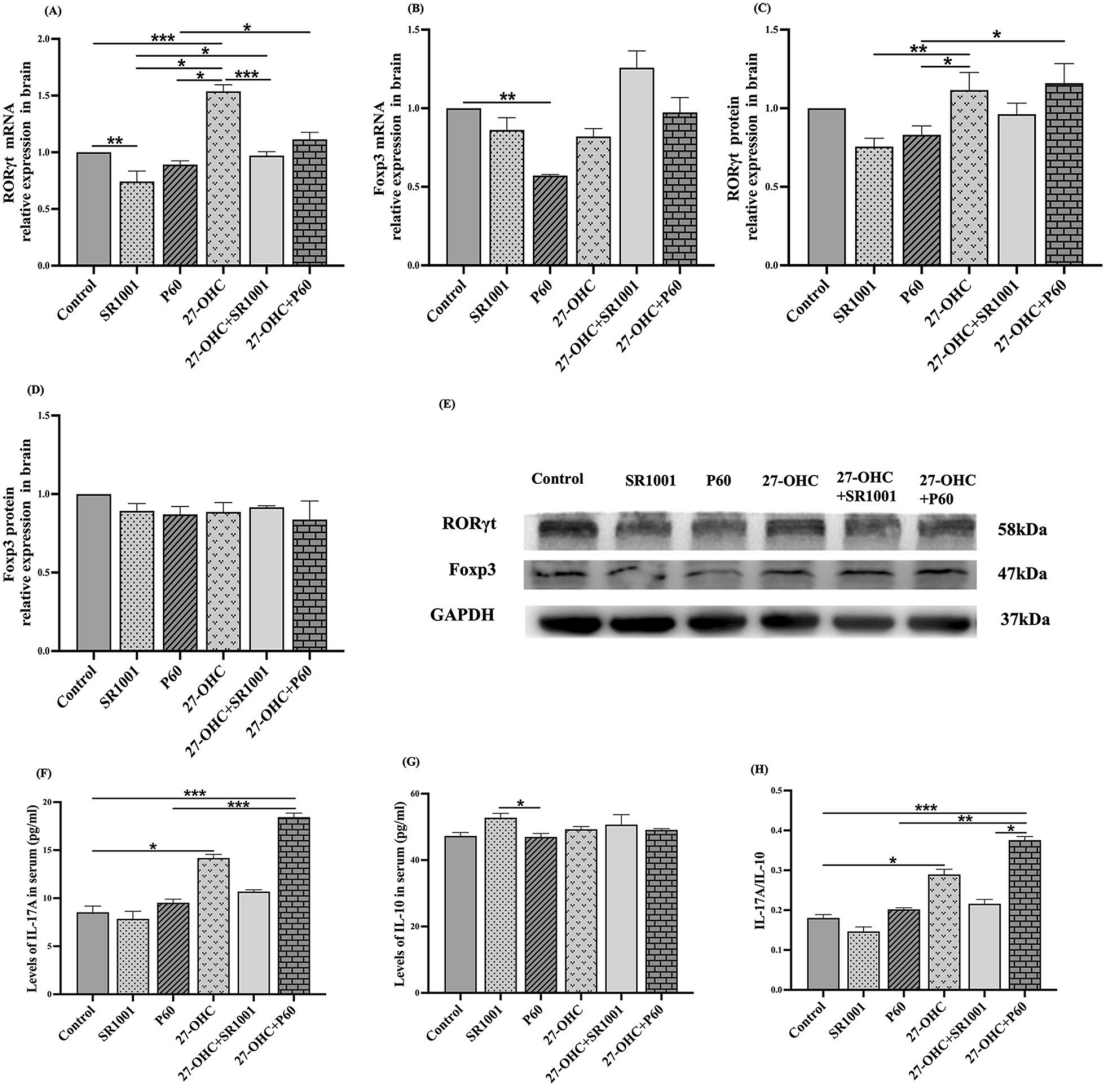
The brain expressions of RORγt and Foxp3 and serum level of IL-17A and IL-10 (*n* = 6 mice/group). **A**, **C** RORγt in the brain; **B**, **D** Foxp3 in the brain; **E** western blot results; **F** IL-17A in the serum; **G** IL-10 in the serum; **H** the ratio of IL-17A/IL-10. All data are presented as mean ± SD. *p < 0.05. **p < 0.01

Second, as the specific transcription factors of Th17/Treg cells, the expression of RORγt and Foxp3 in the brain were detected in this study (Fig. 4A–E). Compared with control group, the expression of RORγt gene in SR1001 group and Foxp3 gene in P60 group decreased by 25.7% (*p* = 0.006) and 13.8% (*p* = 0.001), respectively, suggesting an efficient inhibition of the compounds we used. The expression level of RORγt gene in 27-OHC group was significantly upregulated compared to the control group (*p* < 0.001), while its level decreased sequentially in 27-OHC, 27-OHC + SR1001 and SR1001 group (*p* < 0.001, *p* = 0.012). Besides, the expression level of RORγt gene in 27-OHC + P60 group was higher than that in P60 group (*p* = 0.014).

Then, we detected IL-17A and IL-10 levels in serum (Fig. 4F–H), which were the characteristic cytokines related to Th17/Treg balance. The results showed that the IL-17A level in 27-OHC group was remarkably increased compared with control (*p* = 0.013) group. The IL-17A level in 27-OHC + P60 group was higher than control (*p* < 0.001) and P60 (*p* < 0.001) group. Significant differences in IL-10 level were only observed between SR1001 and P60 group (*p* = 0.049) and the ratio of IL-17A/IL-10 presented similar trend with IL-17A level.

**Fig. 5:**
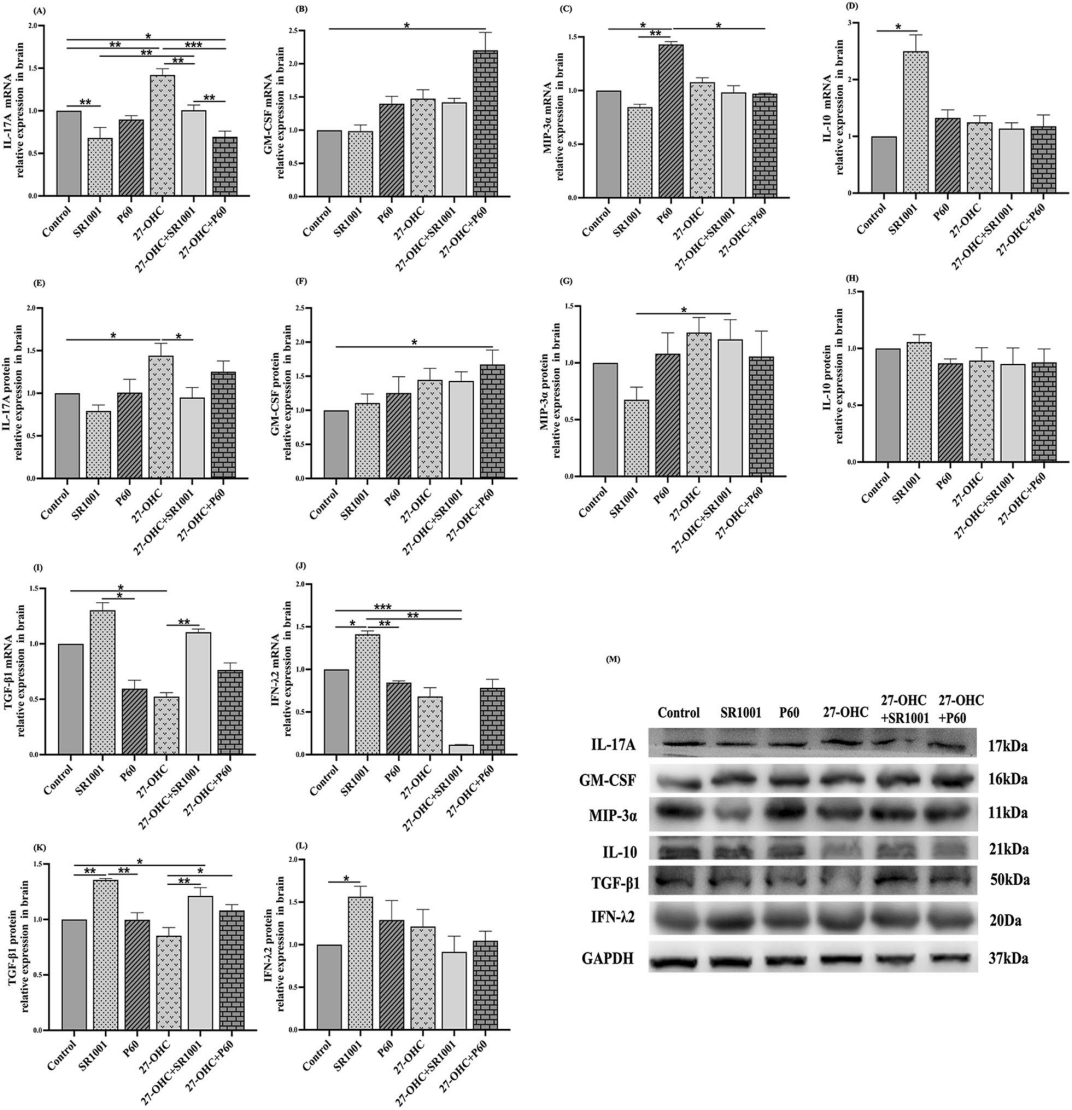
The mRNA and protein expressions of immunomodulatory factors in the brain (*n* = 6 mice/group). **A**, **E** IL-17A; **B**, **F** GM-CSF; **C**, **G** MIP-3α; **D**, **H** IL-10; **I**, **K** TGF-β1; **J**, **L** IFN-λ2; **M** western blot results. All data are presented as mean ± SD. *p < 0.05. **p < 0.01. ***p < 0.001

Moreover, the expression of immunomodulatory factors associated with Th17/Treg balance in the brain was detected. As shown in Fig. 5, compared with the control group, there was higher expression level of IL-17A (gene: *p* = 0.001, protein: *p* = 0.019) and lower TGF-β1 (gene: *p* = 0.025) in 27-OHC-treated mice. In SR1001 group, the expression of IL-17A (gene: *p* = 0.008) was evidently downregulated while IL-10 (gene: *p* = 0.019), TGF-β1 (protein: *p* = 0.001) and IFN-λ2 (gene: *p* = 0.040, protein: *p* = 0.029) were upregulated. When compared with P60 group, the expression of MIP-3α (gene: *p* = 0.001) was decreased while TGF-β1 (gene: *p* = 0.019, protein: *p* = 0.001) and IFN-λ2 (gene: *p* = 0.006) was increased in SR1001 group. In addition, by inhibiting RORγt in 27-OHC-loaded group, the expression level of IL-17A (gene: *p* = 0.001, protein: *p* = 0.011) in 27-OHC + SR1001 group was significantly decreased than that in the single 27-OHC-treated group, but TGF-β1 (gene: *p* = 0.002, protein: *p* = 0.001) was remarkably increased. In contrast, when compared with the SR1001 group, higher IL-17A gene (*p* = 0.007) but lower IFN-λ2 gene (*p* = 0.004) was observed in 27-OHC + SR1001 group.

### Effects of experimental intervention on oxysterols level and metabolic enzymes expression

**Fig. 6:**
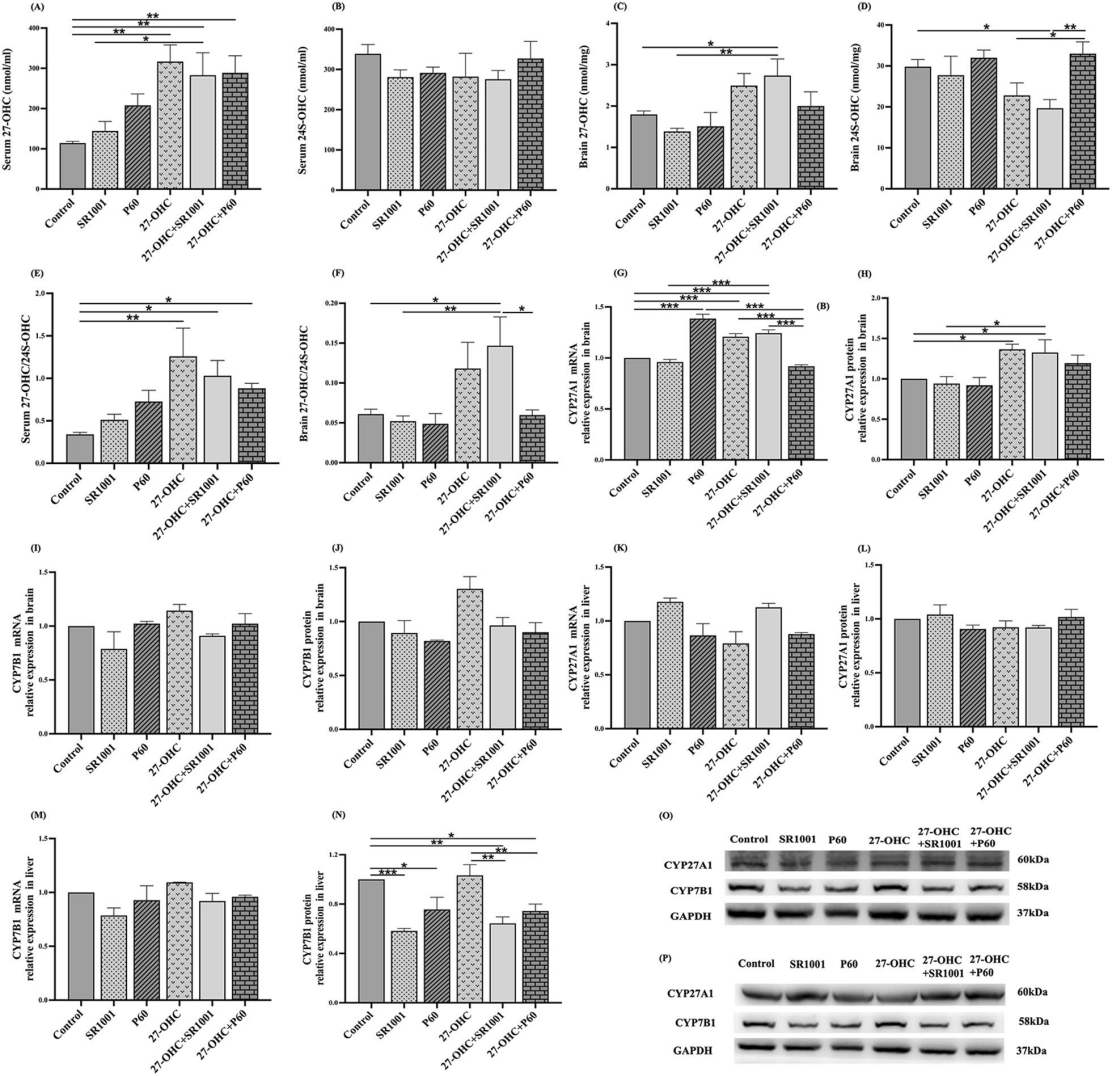
The level of 27-OHC and 24S-OHC and expressions of related metabolic enzymes (*n* = 6 mice/group). **A**, **B** Serum 27-OHC, 24S-OHC; **C**, **D** brain 27-OHC, 24S-OHC; **E** serum 27-OHC/24S-OHC; **F** brain 27-OHC/24S-OHC; **G**, **H** CYP27A1 in the brain; **I**, **J** CYP7B1 in the brain; **K**, **L** CYP27A1 in the liver; **M**, **N** CYP7B1 in the liver; **O** Western blot results in the brain; **P** Western blot results in the liver. All data are presented as mean ± SD. *p < 0.05. **p < 0.01. ***p < 0.001

The levels of 27-OHC and 24S-OHC, the abnormal interplay between which is the main driving force of cholesterol metabolism dysfunction in the brain, are shown in Fig. 6A–F, there were higher 27-OHC level in serum of all the three groups treating with 27-OHC (27-OHC: *p* = 0.002, 27-OHC + SR1001: *p* = 0.007, 27-OHC + P60: *p* = 0.005) compared with the control group. Since no difference was observed in 24S-OHC level, the ratio of 27-OHC/24S-OHC in serum presented similar trend with 27-OHC level. The brain 27-OHC level as well as the 27-OHC/24S-OHC ratio in 27-OHC + SR1001 group were also increased compared with the control (*p* = 0.038, *p* = 0.014) and SR1001 (*p* = 0.006, *p* = 0.008) group. However, the brain 24S-OHC level in 27-OHC + SR1001 group was evidently reduced compared with the control (*p* = 0.029) and 27-OHC + P60 (*p* = 0.006) group.

The expression of CYP27A1 and CYP7B1 in the brain, as the crucial enzymes involved in the synthesis and catabolism of 27-OHC, were further measured (Fig. 6G–J, O). The expression of CYP27A1 in 27-OHC and 27-OHC + SR1001 group was higher than the control mice at both gene (*p* < 0.001, *p* < 0.001) and protein (*p* = 0.020, *p* = 0.034) level. And after treatment with 27-OHC + SR1001, the CYP27A1 level was significantly increased compared with the single SR1001 treatment (gene: *p* < 0.001, protein: *p* = 0.015). The CYP27A1 mRNA in P60 group was remarkably upregulated when compared to control (*p* < 0.001) and 27-OHC + P60 (*p* < 0.001) groups.

Considering that the liver is the main organ for the production of the two enzymes and the metabolism of 27-OHC outside the brain [24], we also measured the expression of CYP27A1 and CYP7B1 in liver (Fig. 6K–N, P). No obvious alteration was found in CYP27A1 at both gene and protein level, so as CYP7B1 mRNA expression. Nevertheless, the expression of CYP7B1 protein in liver was evidently different among the groups. It was worth noting that compared with the control group, the SR1001-treated mice (SR1001: *p* < 0.001, 27-OHC + SR1001: *p* = 0.002) and P60-treated mice (P60: *p* = 0.017, 27-OHC + P60: *p* = 0.014) showed significantly decreased CYP7B1 protein.

### Effects of experimental intervention on the morphology and amyloidosis of brain

**Fig. 7:**
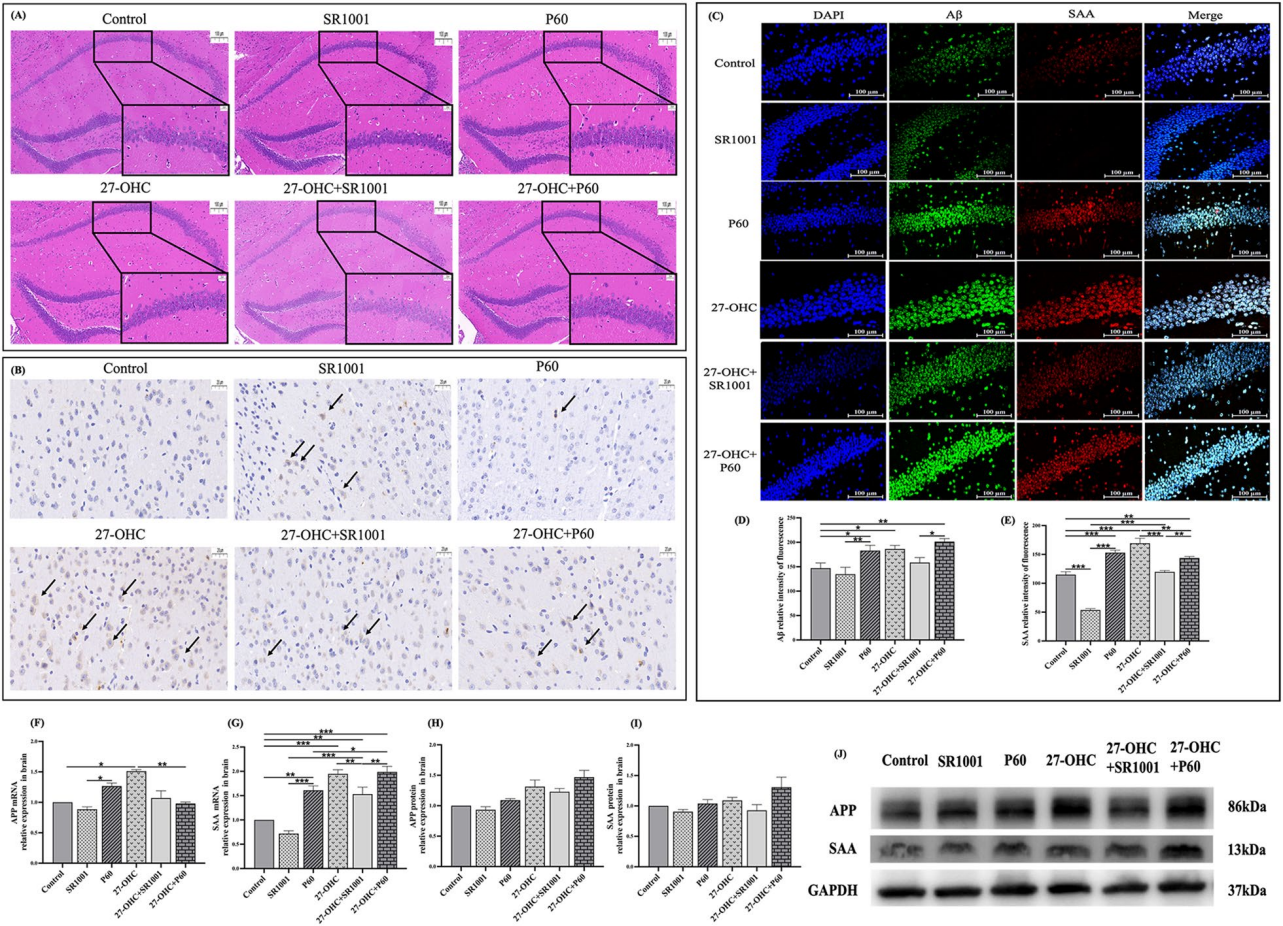
Effects of experimental interventions on brain pathology and amyloidosis in the brain. **A** HE staining of the whole brain (*n* = 3 mice/group, scale bar = 100/20 μm); **B** immunohistochemical staining of Aβ in the cerebral cortex (*n* = 3 mice/group, scale bar = 100/20 μm), black arrows: Aβ deposition; **C** immunofluorescent double staining of Aβ and SAA in the hippocampus (*n* = 3 mice/group, scale bar = 100 μm), nucleus, Aβ and SAA were represented in blue, green and red, respectively; **D**, **E** Aβ and SAA relative intensity of fluorescence; **F**–**I** the mRNA and protein expression of APP and SAA in the brain (*n* = 6 mice/group); **J** Western blot results. All data are presented as mean ± SD. *p < 0.05. **p < 0.01. ***p < 0.001

HE staining was conducted to intuitively assess the effects of treatments on brain histomorphology. As presented in Fig. 7A, the arrangement of neurons in the hippocampal CA1 region was loose and disordered with enlarged intercellular space in the 27-OHC group and 27-OHC + P60 group, along with unclear boundary between cell membranes and nuclear membranes and hyperchromatism of nuclei. However, the brain histomorphology of 27-OHC + SR1001 group was improved compared with that of 27-OHC group, without obvious change in cell number and arrangement.

Immunohistochemical staining was used to observe the Aβ deposition in cerebral cortex of mice and the images are illustrated in Fig. 7B, almost no obvious Aβ deposition was found in control group, while a great deal of Aβ deposition appeared in cerebral cortex of 27-OHC and 27-OHC + P60 group. Conversely, diminished Aβ deposition was observed in the 27-OHC + SR1001 group in comparison with the 27-OHC group.

In addition, immunofluorescent double staining was performed to probe the deposition and specific location of Aβ and SAA proteins in the brain. As expected, images from laser confocal microscopy showed that severe Aβ deposition could be observed in the three 27-OHC-treated group (Fig. 7C) and according to the merge image, Aβ and SAA were co-localized in most areas of accumulation. Compared with control group, the fluorescence intensity of Aβ and SAA was evidently enhanced in 27-OHC (*p* = 0.019, *p* < 0.001) and P60 (*p* = 0.029, *p* < 0.001) mice, and the treatment of 27-OHC + P60 also caused higher level of Aβ and SAA (*p* = 0.003, *p* = 0.001). Moreover, 27-OHC + SR1001 significantly reduced the fluorescence intensity of SAA compared with the 27-OHC group (*p* < 0.001). Treating with SR1001 induced lower SAA fluorescence intensity than that of the control (*p* < 0.001), P60 (*p* < 0.001) and 27-OHC + SR1001 (*p* < 0.001) mice (Fig. 7D–E).

Further, as APP and SAA are pivotal amyloid precursors participated in amyloidosis, their expressions in the brain were measured (Fig. 7F–J). The expression of APP (*p* = 0.011) and SAA (*p* < 0.001) mRNA were upregulated in the 27-OHC group compared with control group, and the expression of the two genes in P60 group was higher than that in the SR1001 group (APP: *p* = 0.032, SAA: *p* < 0.001). As for the expression of SAA gene, the level of which in 27-OHC + SR1001 (*p* = 0.002) and 27-OHC + P60 (*p* = 0.005) group was significantly increased compared with control group. And by adding 27-OHC injection to the mice with SR1001/P60 treatment, the expression of SAA gene in the brain was significantly increased (27-OHC + SR1001: *p* < 0.001, 27-OHC + P60: *p* < 0.001).

## Discussion

**Fig. 8:**
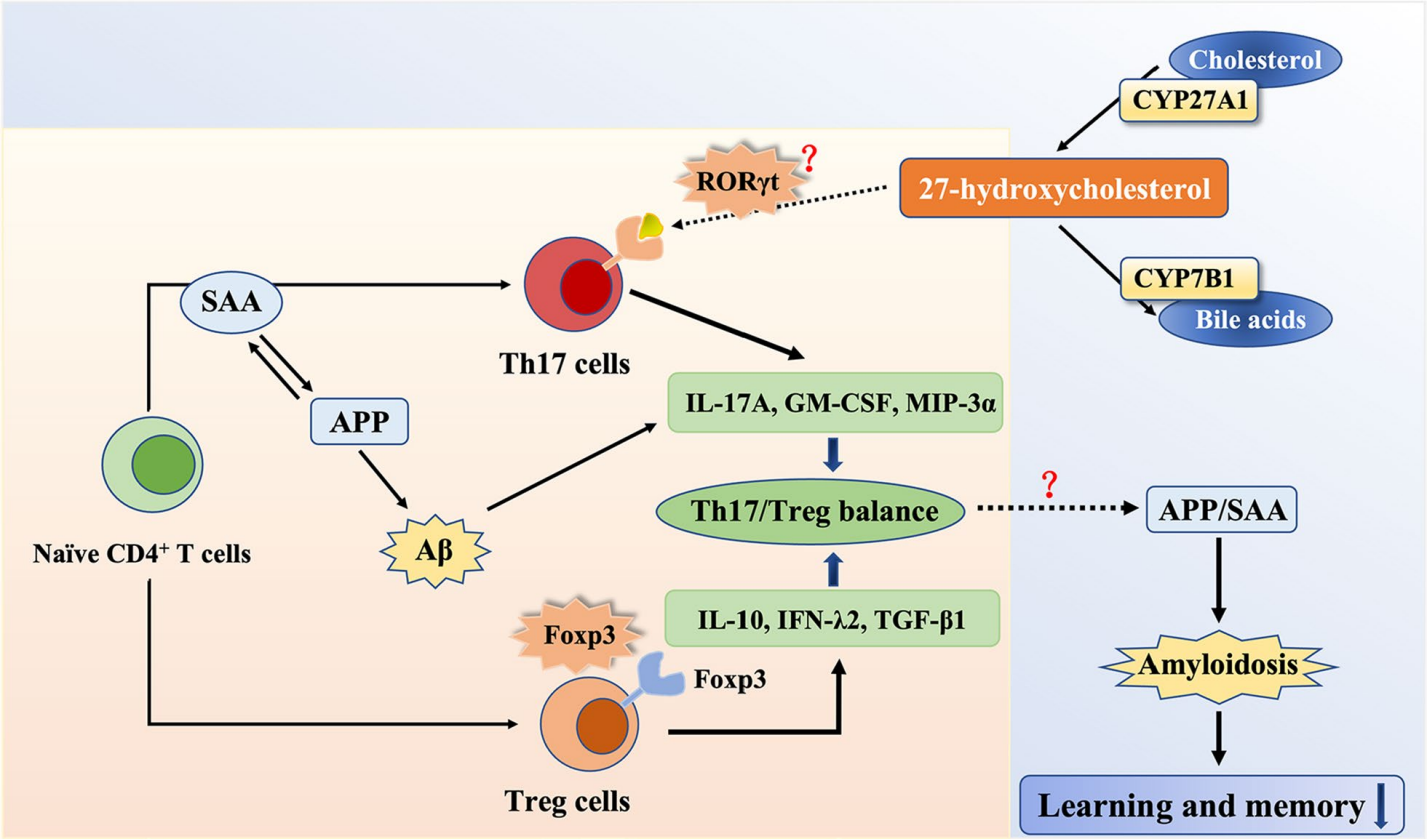
Schematic displays effects of 27-hydroxycholesterol on cognitive deficits by regulating RORγt and disturbing Th17/Treg balance

The homeostasis of oxysterols changes in cognitive deficits and evidence shows that they have broad functions in this pathological process [25]. This study focuses on 27-OHC, which has been reported in our previous research as an independent risk factor for MCI [5]. Since 27-OHC is reported to be a potent agonist of RORγt, whether 27-OHC regulates the expression of RORγt and the subsequent Th17 signaling deserves further investigation. In this study, we found significantly high level of 27-OHC and altered homeostasis of Th17-related cytokines in serum of MCI individuals. The potential mechanism by which 27-OHC affecting cognitive function might be that 27-OHC disturbs Th17/Treg balance and the related immune responses by regulating RORγt, which aggravates the amyloidosis and leads to learning and memory decline (Fig. 8).

Increasing evidence highlights the early and substantial involvement of immunological mechanisms in the brain of cognitive impairment individuals [26]. Although the mechanism by which Th17 cells contribute to cognitive decline is unclear, considerable evidence has reported that Th17 phenotype and its characteristic cytokines are different in AD patients and mouse models [27]. For example, Levite et al. found that significantly high levels of Th17-specific (IL-17 and IL-21) and Th17-related (TNF-α) cytokines were produced by activated Th17 cells, which induced neuron loss and blood–brain barrier dysfunction [28]. In this study, higher IL-17A, IL-12p70, IL-23, GM-CSF, MIP-3α and TNF-α but lower IL-13, IL-28A and TGF-β1 were found in serum of MCI individuals. And results of logistic regression supported that IL-17A, IL-12p70, GM-CSF and TNF-α could increase the risk of MCI while IL-13 and TGF-β1 showed the opposite effects.

Studies indicated that excessive cholesterol in serum led to the immunological synapse formation and T cell receptor (TCR) clustering increase, facilitating TCR-dependent signaling and the related immune responses [29]. Given the current work that linking oxysterols to cholesterol metabolism and cognitive function, we also detected the levels of four oxysterols in serum of MCI individuals. Yet, no significant correlation was found between IL-17A and oxysterols, which might be due to the small sample size and the differences in population characteristics of this study. The real relationship between oxysterols, especially 27-OHC, and neuroimmune mechanisms in cognitive decline, and whether overloading of 27‐OHC in vivo disturbing Th17-related cytokines and learning and memory, are still unclear. Therefore, C57BL/6J mice were used for further exploration.

It has been well-established that the hydroxyl group at carbon 27 of oxysterols is needed for RORγt agonism. CYP27A1-knockout decreased the level of 27-OHC, reduced Th17 cells differentiation and IL-17 production, which was also observed in RORγt knockout mice, indicating that 27-OHC might impact the production of IL-17 via a RORγt-dependent manner [14]. It is worth exploring how 27-OHC influences IL-17 signaling and brain functions. Here, 27-OHC treatment increased the proportion of Th17 cells in PBMCs and hippocampus, high level of IL-17A was observed in both serum and brain, but the expression of TGF-β1 in the brain was downregulated. In contrast, along with the significant downregulation of RORγt gene in 27-OHC + SR1001 group, the Th17 proportion and IL-17A level were reduced. Collectively, our results indicated that 27-OHC-induced Th17/Treg imbalance and abnormal immune responses might be mediated by regulating RORγt.

In addition, the 27-OHC-treated mice showed significantly poor performance in MWM test, but inhibiting of RORγt and Foxp3 induced a tendency to attenuate and enhance the effects of 27-OHC, respectively. Helena et al. reported an accumulation of IL-17 in the brain, meninges and cervical lymph nodes of 3xTg-AD models at early stage of disease, while neutralizing IL-17 delayed the amyloidosis-related splenomegaly and short-memory deficits [30]. Tahmasebinia et al. found an imbalance of Th17-related cytokines in the brain of AD model rats induced by Aβ_1-42_, manifested by increased RORγt, IL-17 and IL-23, while decreased TGF-β and IL-35 [31]. These studies are consistent with our results, confirming the possible involvement of Th17 cells and IL-17A in AD pathology. Moreover, our results also support the promotion of 27-OHC on RORγt, suggesting that 27-OHC causes learning and memory decline by regulating RORγt and disturbing Th17/Treg balance and the related immune responses.

It has been widely recognized that production of the neurotoxic Aβ from APP proteolysis is the pivotal step in the progression of AD. Nevertheless, Aβ is not the only factor affecting the cognitive function and brain pathology [32]. SAA, as the precursor of AA amyloidosis, has been reported to be sequentially elevated in the serum of normal, MCI and AD individuals [33]. To establish the relation between amyloidosis and cognitive decline, we detected the expression level of key precursor proteins in the brain. The results showed that in 27-OHC-treated mice, both APP and SAA gene were upregulated, and the fluorescence intensity of Aβ and SAA in the hippocampus was increased with significant colocalization. Although SAA is synthesized primarily in the liver, Ryoo et al. found that liver-derived SAA could migrate into the brain and exacerbate Aβ accumulation and glial activation in SAA transgenic mice. Further, they found that the APP and SAA double transgenic mice presented relatively intense apoptosis and immune responses in the brain, concluding that SAA enhanced the neuroinflammation in Aβ abundant condition and induced severe cognitive deficits compared with the single transgene of APP [34, 35]. Therefore, we speculate that 27-OHC regulates the Th17/Treg-related immune responses, which further affects the brain amyloidosis and cognitive function.

## Conclusion

Overall, our study reveals a link between Th17-related cytokines spectrum and cognitive function in MCI population. Through in vivo experiments with C57BL/6J mice, we demonstrate that 27-OHC disturbs Th17/Treg balance by regulating the indispensable nuclear factor-RORγt, which increases the expression of amyloid precursors in the brain, resulting in the amyloid deposition and brain morphological damage and the eventual cognitive decline. This study reveals a new potential therapeutic target for the inappropriate immune responses in AD, but further studies are warranted to better evaluate the molecular mechanisms of Th17/Treg imbalance after 27-OHC treatment.

## Data Availability

The datasets used and/or analyzed during the current study are available from the corresponding author on reasonable request.

## Abbreviations

24S-OHC: 24S-hydroxycholesterol
27-CA: 3β-Hydroxy-5-cholestenoic acid
27-OHC: 27-Hydroxycholesterol
7-HOCA: 7α-Hydroxy-3-oxo-4-cholestenoic acid
AA: Amyloid A
AD: Alzheimer’s disease
APP: Amyloid precursor protein
Aβ: Amyloid-β
BMI: Body mass index
FDI: Frequency identification index
Foxp3: Forkhead box protein P3
GM-CSF: Granulocyte–macrophage colony-stimulating factor
IFN-λ2: Interferon-λ2
MCI: Mild cognitive impairment
MIP-3α: Macrophage inflammatory protein 3α
MMSE: Mini-Mental State Examination
MoCA: Montreal Cognitive Assessment
MWM: Morris water maze
NORI: Novel object recognition index
PBMCs: Peripheral blood mononuclear cells
RORγt: Retinoic acid-related orphan receptor γt
SAA: Serum amyloid A
TDI: Time discrimination index
TGF-β1: Transforming growth factor β1
Th17: T helper 17
Treg: Regulatory T
